# Disruption of the Circadian Clock in Mice Increases Intestinal Permeability and Promotes Alcohol-Induced Hepatic Pathology and Inflammation

**DOI:** 10.1371/journal.pone.0067102

**Published:** 2013-06-18

**Authors:** Keith C. Summa, Robin M. Voigt, Christopher B. Forsyth, Maliha Shaikh, Kate Cavanaugh, Yueming Tang, Martha Hotz Vitaterna, Shiwen Song, Fred W. Turek, Ali Keshavarzian

**Affiliations:** 1 Center for Sleep and Circadian Biology, Department of Neurobiology, Northwestern University, Evanston, Illinois, United States of America; 2 Northwestern University Feinberg School of Medicine, Chicago, Illinois, United States of America; 3 Division of Digestive Diseases and Nutrition, Department of Internal Medicine, Rush University Medical Center, Chicago, Illinois, United States of America; 4 Department of Biochemistry, Rush University Medical Center, Chicago, Illinois, United States of America; 5 American Society for Clinical Pathology, Chicago, Illinois, United States of America; 6 Department of Pharmacology, Rush University Medical Center, Chicago, Illinois, United States of America; 7 Department of Molecular Biophysics and Physiology, Rush University Medical Center, Chicago, Illinois, United States of America; 8 Division of Pharmacology, Utrecht Institute for Pharmaceutical Sciences, Faculty of Science, Utrecht University, Utrecht, The Netherlands; University of Chicago, United States of America

## Abstract

The circadian clock orchestrates temporal patterns of physiology and behavior relative to the environmental light:dark cycle by generating and organizing transcriptional and biochemical rhythms in cells and tissues throughout the body. Circadian clock genes have been shown to regulate the physiology and function of the gastrointestinal tract. Disruption of the intestinal epithelial barrier enables the translocation of proinflammatory bacterial products, such as endotoxin, across the intestinal wall and into systemic circulation; a process that has been linked to pathologic inflammatory states associated with metabolic, hepatic, cardiovascular and neurodegenerative diseases – many of which are commonly reported in shift workers. Here we report, for the first time, that circadian disorganization, using independent genetic and environmental strategies, increases permeability of the intestinal epithelial barrier (i.e., gut leakiness) in mice. Utilizing chronic alcohol consumption as a well-established model of induced intestinal hyperpermeability, we also found that both genetic and environmental circadian disruption promote alcohol-induced gut leakiness, endotoxemia and steatohepatitis, possibly through a mechanism involving the tight junction protein occludin. Circadian organization thus appears critical for the maintenance of intestinal barrier integrity, especially in the context of injurious agents, such as alcohol. Circadian disruption may therefore represent a previously unrecognized risk factor underlying the susceptibility to or development of alcoholic liver disease, as well as other conditions associated with intestinal hyperpermeability and an endotoxin-triggered inflammatory state.

## Introduction

Mammalian circadian organization consists of a cell-autonomous molecular pacemaker active in nearly all cells of the body that drives the expression of thousands of genes (i.e., “clock-controlled genes”) and regulates numerous biochemical and physiological rhythms [1]. Within the gastrointestinal tract, circadian clock genes are expressed [2], and contribute to the regulation of colonic motility, nutrient absorption and cell proliferation [3], [4], [5]. Not surprisingly, circadian disruption (e.g., shift work) has been linked to gastrointestinal disease, including exacerbated irritable bowel syndrome symptoms and increased risk for developing colorectal cancer [6], [7], and gastrointestinal complaints commonly accompany jet lag [8]. Furthermore, we recently demonstrated that chronic circadian disruption worsened dextran sodium sulfate (DSS)-induced colitis in mice: repeated phase shifts of the light:dark (LD) cycle accelerated disease onset, worsened severity of histopathological damage, exacerbated inflammation and increased mortality [9]. This finding provided compelling evidence that perturbation of the circadian clock renders intestinal epithelial cells vulnerable to injury. Similarly, sleep deprivation, both acute and chronic, augments inflammation and pathology in DSS-treated mice [10]. Taken together, these observations indicate that circadian organization is important for optimal gastrointestinal physiological function and highlight the relevance of disruption of circadian rhythms for pathologies within the gastrointestinal tract, in particular those involving intestinal epithelial barrier integrity.

Maintenance of intestinal epithelial barrier integrity is essential for protection from proinflammatory intestinal luminal contents, such as bacterial endotoxins (i.e., lipopolysaccharide, LPS) [11]. The mucosal barrier is maintained by a complex network of interacting proteins, including tight junction, adherens junction and desmosome proteins [11]. Disruption of the barrier (i.e., gut leakiness) is associated with numerous diseases, including metabolic syndrome, diabetes, cardiovascular disease, amyotrophic lateral sclerosis, Parkinson's disease and alcoholic liver disease [12], [13], [14], [15], [16], [17], [18]. Thus, intestinal hyperpermeability is a clinically relevant pathology and the development of gut leakiness may represent a biologically meaningful therapeutic target for numerous diseases. However, the factors contributing to the onset of intestinal hyperpermeability are poorly understood.

Several lines of evidence suggest a potential role for circadian clock genes in the regulation of intestinal barrier function. First, many diseases associated with circadian disruption exhibit increased gut leakiness [19], [20], [21]. Second, the primary mechanism of DSS-induced colitis in rodents is impaired intestinal barrier integrity, and we have shown that circadian disruption exacerbates colitis in DSS-treated mice [9]. Third, our recent study [22] implicates circadian clock genes in the regulation of intestinal epithelial barrier integrity *in vitro* (i.e., Caco-2 monolayers, a human intestinal epithelial cell line used to model barrier function): siRNA knockdown of the canonical circadian genes *Clock* and *Per2* blocks alcohol-induced increases in Caco-2 layer permeability [22]. However, direct *in vivo* evidence to support the hypothesis that disruption of circadian homeostasis alters intestinal barrier function is lacking.

The aim of the current study was to fill this gap in our knowledge and determine whether disruption of circadian organization causes gut leakiness and promotes pathological conditions associated with gut leakiness and LPS-mediated tissue injury. We found that circadian disruption in mice, genetically *via* homozygous *Clock^Δ19/Δ19^* mutation [23], [24] or environmentally *via* chronic phase shifts of the LD cycle, significantly increased intestinal permeability. In addition, we superimposed chronic alcohol consumption with circadian disruption in order to determine the impact on intestinal barrier integrity in the context of an environmental challenge (i.e., chronic alcohol exposure), as chronic alcohol consumption is a well-established model of inducing intestinal hyperpermeability, endotoxemia and inflammatory hepatic pathology [14]. Both genetic and environmental circadian disruption promoted alcohol-induced gut leakiness and hepatic pathology, possibly through a mechanism involving, at least in part, altered regulation of the tight junction protein occludin.

## Materials and Methods

### Ethics Statement

All mice were housed and handled in accordance with federal animal welfare guidelines and in compliance with the Public Health Service Policy on Humane Care and Use of Laboratory Animals (2002) and the Guide for the Use and Care of Laboratory Animals (8^th^ Edition). All experiments were reviewed and approved prior to being conducted by the Institutional Animal Care and Use Committees of Northwestern University (Animal Study Protocol #2010–2186) and Rush University Medical Center (Animal Study Protocol #10–083).

### Mice and Housing

Studies at Northwestern University utilized *Clock^Δ19/Δ19^* mutant (C57BL/6J coisogenic [24]) and wild-type littermate mice obtained from the breeding colony maintained at the university. Individually housed young adult (7–9 week) males were used for all studies. Mice were maintained on a constant 12 hour light:12 hour dark light cycle (12∶12 LD) with lights on at 6am Central Daylight Time and lights off at 6pm Central Daylight Time. Locomotor activity was measured throughout the experiment using infrared beam breaks. Briefly, three infrared beams are projected across each cage and activity is recorded when a beam is broken. Food intake and body weight were recorded for the duration of the experiment.

Studies at Rush University Medical Center utilized wild-type C57BL/6J mice (B6 hereafter; The Jackson Laboratory, Bar Harbor, ME). Young adult (6–8 week old) mice arrived and were individually housed in cages stored in ventilated, light-tight cabinets as described above. Mice were acclimated to the animal facility for one week prior to initiating the study. Locomotor activity, food intake and body weight were measured throughout the experiment.

By convention, for animals maintained on a 12∶12 LD cycle, the time of the onset of light is referred to as Zeitgeber time (ZT)0, while the time of lights off is referred to as ZT12. The cages were located in ventilated, light-tight cabinets as described previously [25].

### Circadian Disruption

#### Genetic

Mice homozygous for the *Clock^Δ19^* mutation [23], [24] were used to study genetic disruption of circadian rhythms (conducted at Northwestern University). The dominant negative *Clock^Δ19^* mutation [26], [27] was produced by chemical mutagenesis of C57BL/6J mice [24], making this line coisogenic. This model is well-validated and widely used to explore the link between circadian rhythms and many biological processes, including metabolism [28], reproductive physiology [29], affective behavior [30] and sleep [31], among others. Experimental subjects were all progeny of a ^Δ19^/+ × ^Δ19^/+ mating so that wild-type littermates could be used as controls. All mice were genotyped from PCR of DNA isolated from tail tip biopsies collected at the time of weaning (21 days).

#### Environmental

**Figure 1:**
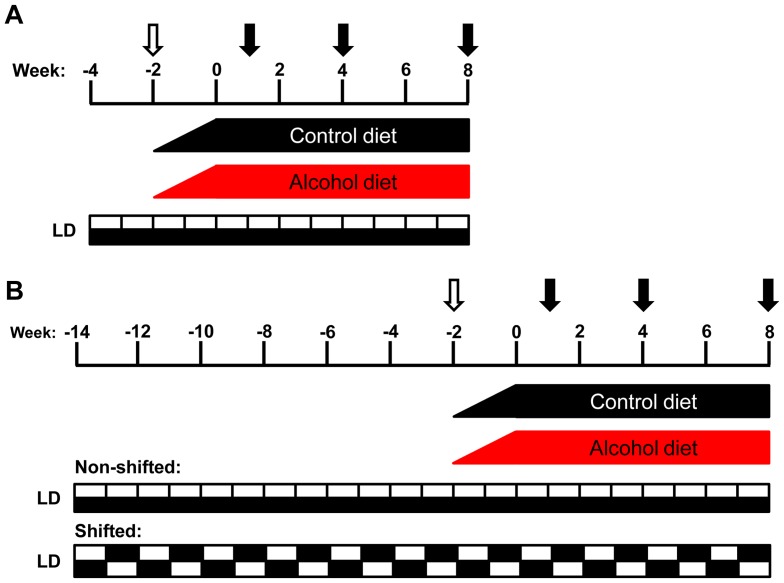
Models of circadian disruption and chronic alcohol consumption experimental protocols. (**A**) Young adult (7–9 week) male *Clock^Δ19/Δ19^* mutant mice and wild-type littermates (C57BL/6J coisogenic) were individually housed and maintained on a constant 12∶12 LD cycle for the duration of the experiment. Mice of each genotype were randomly assigned to receive either the alcohol-containing or isocaloric control diet, resulting in four experimental groups: wild-type dextrose control (WTD), wild-type alcohol (WTA), *Clock^Δ19/Δ19^* mutant dextrose control (CD) and *Clock^Δ19/Δ19^* mutant alcohol (CA). There was a gradual two week increase in alcohol concentration (open arrow; weeks −2–0; 0–29% total calories from alcohol) followed by eight weeks on the full alcohol diet (weeks 0–8; 29% total calories from alcohol; 4.5% v/v). Intestinal permeability was measured at weeks 1, 4 and 8 (closed arrows). At the end of week 8, mice were euthanized at ZT6 and tissues were collected for analyses. (**B**) Young adult (7–9 week) male C57BL/6J mice were individually housed. Mice were randomized into one of two light schedules: non-shifted mice were maintained on a constant 12∶12 LD cycle and shifted mice were subjected to a weekly 12 hour phase shift of the LD cycle for 12 weeks. Shifted and non-shifted mice were randomized into one of two diet treatments: alcohol or control, resulting in four experimental groups: non-shifted control diet (NSD), non-shifted alcohol (NSA), shifted control diet (SD) and shifted alcohol (SA). There was a gradual two week increase in alcohol as described in (A). Intestinal permeability was measured at weeks 1, 4 and 8 (closed arrows). At the end of week 8, mice were euthanized in groups every four hours across the diurnal cycle (at ZT0, ZT4, ZT8, ZT12, ZT16 and ZT20).

Environmental disruption of circadian rhythms was achieved using a once weekly 12 hour phase shift in the LD cycle (conducted at Rush University Medical Center). Mice randomized into the shifted groups were exposed to weekly 12 hour phase shifts of the LD cycle for three months prior to the initiation of the liquid diet protocol (described below, Figure 1) and continued for the duration of the 10 week experiment. Mice randomized into the non-shifted groups were maintained on a constant 12∶12 LD cycle for the entire experiment.

### Chronic Alcohol Consumption Protocol (Figure 1)

Both genetic and environmental models of circadian disruption used the Nanji diet alcohol protocol [32], [33], [34], [35], which consisted of a two week introduction and gradual increase in alcohol dose, followed by eight weeks on the full alcohol concentration (29% of total calories, 4.5% v/v; Figure 1). Control mice were fed an isocaloric liquid diet in which the calories from alcohol were replaced with dextrose. The components of the liquid Nanji diet include: mineral mix, vitamin mix, choline bitartrate, d-L-methionine, lactalbumin, xanthan gum, dextrose (all obtained from Dyets, Inc., Bethlehem, PA), fish oil (from menhaden), ethanol (both from Sigma, St. Louis, MO) and Hershey's Lite Chocolate syrup. The caloric composition of the diet was: 36% protein, 29% carbohydrate/alcohol and 35% fat. The liquid diet for each group was prepared fresh daily and provided to mice in individual specialized graduated sipper tubes (Bio-Serv, Frenchtown, NJ) to allow for monitoring of daily food intake.

### In Vivo Intestinal Permeability Testing

*In vivo* assessment of intestinal permeability was conducted as described previously [13], [36] using a well-validated model to determine permeability across the epithelial barrier in the small intestine and colon [37], [38]. Briefly, mice were fasted for eight hours prior to the test, which was performed at ZT0 (i.e., light onset). A 200 μL solution containing lactulose (3.2 mg), sucrose (0.45 mg), sucralose (0.45 mg) and mannitol (0.9 mg) was administered via oral gavage, after which 2 mL 0.9% saline was administered subcutaneously to promote urine production. Urine produced over five hours was collected and the total volume was recorded. The five hour time window was selected due to transit time through the intestine and thus captures the permeability across the entire intestine, including the colon. Intestinal permeability was determined by measuring urinary sugar concentration using gas chromatography, enabling calculation of the amount of orally administered sugar excreted in the urine over five hours. Intestinal permeability was measured 1, 4 and 8 weeks after initiating the full (i.e., 29%) alcohol concentration in the diet.

### Tissue Collection

At the conclusion of the experiment, mice were euthanized by conscious decapitation. Proximal colon and liver tissues were harvested for analyses. Samples were either snap-frozen in liquid nitrogen or placed into RNALater (Qiagen, Valencia, CA) and frozen. Blood was collected and allowed to clot at room temperature prior to centrifugation for serum collection. All tissue and serum samples were stored at −80°C until use. *Clock^Δ19/Δ19^* mutant and wild-type littermate tissues were collected at ZT6 (i.e., 6 hours after lights on). Tissues from shifted and non-shifted B6 mice were collected every four hours across the diurnal cycle, starting at ZT0 (i.e., ZT0, ZT4, ZT8, ZT12, ZT16 and ZT20).

### Assessment of Serum Alcohol Levels

Serum collected at the time of euthanasia was used to determine serum alcohol levels by head space chromatography as described previously [39]. Briefly, serum was precipitated with perchlonic acid/thiourea containing 1 mM 2-propanol, which was included as an internal standard. Samples were heated to 67°C and the vapor phase was quantified for alcohol concentrations using a Perkin-Elmer gas chromatograph. Data are expressed as mg/dL.

### Assessment of Endotoxemia

#### Lipopolysaccharide (LPS)

LPS, a component of the outer membrane of Gram-negative bacteria, is an indicator of intestinal leakiness. Serum collected at the time of euthanasia was used to measure systemic LPS levels using an LPS ELISA kit (MBS-722939; MyBioSource, San Diego, CA) according to the manufacturer's instructions. Data are expressed as pg/mL.

#### LPS binding protein (LBP)

LBP is a type 1 acute phase protein that is constitutively produced by the liver and rapidly upregulated during acute phase responses [40]. LBP binds LPS to facilitate immune responses in conjunction with cell-surface pattern recognition receptors and is used as an indicator of LPS exposure. Serum collected at the time of euthanasia was used to measure systemic LBP levels using an LBP ELISA kit (HK205; Hycult Biotech, Plymouth Meeting, PA) according to the manufacturer's instructions. Data are expressed as EU/mL.

#### LBP mRNA

LBP mRNA was measured in liver samples prepared as tissue homogenates using Affymetrix lysis buffer and processed according to the manufacturer's instructions. mRNA levels were determined using a Luminex platform-based custom multiplex bead array (Affymetrix, Inc., Santa Clara, CA). Expression was normalized to the housekeeping gene RPLPO. Data are expressed as mean fluorescent intensity (MFI).

### Analysis of Tight Junction Protein Occludin

Occludin is a protein that is critical for the maintenance of intestinal barrier integrity, thus occludin levels were measured as a potential mechanistic contributor to increased intestinal permeability.

#### Protein

Proximal colon tissues were prepared as tissue homogenates and assayed in an Occludin ELISA kit (E92228MU; USCN Life Science, Inc., Houston, TX) according to the manufacturer's instructions. To assess cytosol *vs.* membrane levels of occludin, the proximal colon tissue samples were homogenized in PBS and, following centrifugation, the supernatant was removed and the pellet was re-suspended in PBS-containing detergent. This procedure allows for differentiation of cytosol (supernatant) *vs*. membrane (re-suspended pellet) occludin levels.

#### mRNA

Proximal colon tissues were prepared as tissue homogenates using Affymetrix lysis buffer and processed according to the manufacturer's instructions. mRNA expression was determined using a Luminex platform-based custom multiplex bead array (Affymetrix, Inc., Santa Clara, CA). Occludin mRNA expression was normalized to the housekeeping gene RPLPO. Data are expressed as mean fluorescent intensity (MFI).

### Liver Pathology

#### Liver fat

Liver fat was measured gravimetrically at the time of euthanasia and normalized to body weight. The liver/body weight ratio was used to approximate liver fat content.

#### Histology

Formalin-fixed liver was stained with hematoxylin & eosin (H&E). Blinded assessment of samples was conducted by a gastrointestinal pathologist (SS). Histological analyses, including steatosis, inflammation, ballooning degeneration and the presence of acidophil bodies, were scored according to the following criteria: Steatosis: severity was scored as percent hepatocyte involvement (0 = <5%, 1 = 5–33%, 2 = 34–66%, 3 = >67%), corresponding to the fraction of lipid-containing hepatocytes. Inflammation: severity was scored based on the number of inflammatory foci per 200× field (0 = no foci, 1 = 1 focus, 2 = 2–4 foci, 3 = >4 foci). Ballooning degeneration: scored based on the presence and frequency of ballooned cells (0 = none, 1 = few, 2 = prominent/many), as an indication of hepatocyte injury. Acidophil body presence: estimated by the presence and frequency of acidophil bodies (0 = absent, 1 = focal apoptosis (few acidophil bodies), 2 = many acidophil bodies, 3 = confluent necrosis), corresponding to injured hepatocytes demonstrating a feature of programmed cell death. These markers (steatosis, inflammation, ballooning degeneration and acidophil bodies) were selected because they are all well-established markers of steatohepatitis [41], [42], [43], [44].

### Statistical Analysis

All data are shown as mean ± standard error of the mean (SEM). Statistical evaluation of data was performed using Microsoft Excel or NCSS (Kayesville, UT) software. Individual pairwise comparisons, such as *a priori* tests of baseline differences between genotypes or light schedule (i.e., shifted *vs*. non-shifted), were done by t-test. Otherwise, data were examined by two-way analysis of variance (ANOVA) so that the effects of circadian disruption and diet could be identified separately, as well as the interactions between the two. For data obtained from samples collected at different ZTs, three-way ANOVA was utilized to identify significant time-of-day effects in addition to diet and light schedule effects. Where appropriate, the Tukey-Kramer Multiple-Comparison and Fisher's LSD Multiple-Comparison tests were used for *post-hoc* identification of pairwise group differences.

## Results

### Circadian Disruption – Genetic model

**Figure 2:**
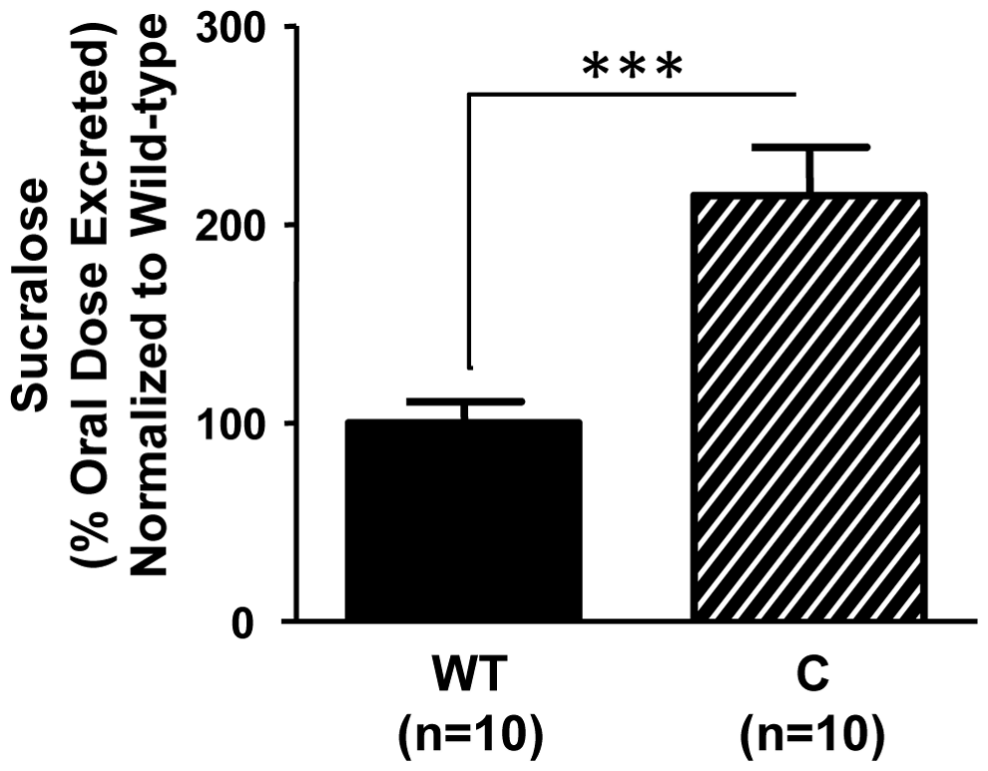
Genetic disruption of circadian organization increases intestinal permeability. Young adult (7–9 week) male *Clock^Δ19/Δ19^* mutant mice (C, n = 10) exhibited increased intestinal permeability, as measured by the urinary excretion of orally administered sucralose, a non-metabolized, non-absorbed sugar, compared to wild-type littermates (WT, n = 10). ***p<0.001, Student's *t*-test.

Using *Clock^Δ19/Δ19^* mutant mice as a genetic model of circadian disorganization [23], [24], we observed that animals homozygous for the *Clock^Δ19^* mutation exhibited significantly increased intestinal permeability, as measured by the urinary excretion of orally administered non-metabolized, non-absorbed sugars (Figure 2; p<0.001, Student's t-test). Five hour urinary sucralose levels were significantly higher in *Clock^Δ19/Δ19^* mutant mice (2.067±0.2% oral dose excreted) than wild-type littermates (0.962±0.1% oral dose excreted). However, there were no significant differences in urinary sucrose (marker of gastroduodenal permeability), mannitol or lactulose (markers of small bowel permeability) between *Clock^Δ19/Δ19^* mutant and wild-type mice (data not shown), indicating that genetic disruption of circadian organization primarily impacts colonic permeability.

**Figure 3:**
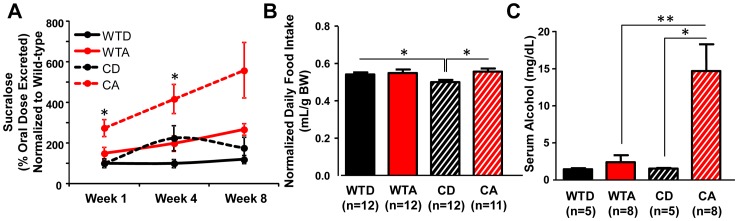
Genetic disruption of circadian organization promotes alcohol-induced intestinal hyperpermeability. (**A**) Urinary sucralose excretion was measured as an index of colon permeability. Alcohol-fed *Clock^Δ19/Δ19^* mutant mice (CA, n = 8–14/week) exhibited increased intestinal permeability compared to alcohol-fed wild-type mice (WTA, n = 8–11/week), control-fed *Clock^Δ19/Δ19^* mutants (CD, n = 8–10/week) and control-fed wild-type mice (WTD, n = 8–9/week). Overall, significant effects of diet (p<0.01) and genotype (p<0.01) were observed. *p<0.05, three-way ANOVA followed by *post-hoc* Tukey-Kramer Multiple-Comparison test. (**B**) Average daily diet intake (normalized to body weight) over the duration of the experiment. Alcohol-fed *Clock^Δ19/Δ19^* mutant mice (CA) did not consume more alcohol than wild-type littermates (WTA). *p<0.05, two-way ANOVA followed by *post-hoc* Tukey-Kramer Multiple-Comparison test. (**C**) Serum collected at ZT6 was assessed for alcohol levels using gas chromatography. Alcohol-fed *Clock^Δ19/Δ19^* mutant mice (CA) exhibited significantly increased serum alcohol compared to alcohol-fed wild-type mice (WTA) and control diet-fed *Clock^Δ19/Δ19^* mutants (CD). *p<0.05, **p<0.01; two-way ANOVA followed by *post-hoc* Tukey-Kramer Multiple-Comparison test.

Utilizing chronic alcohol consumption as a model to induce intestinal hyperpermeability [14], we sought to determine whether genetic perturbation of the circadian system by the *Clock^Δ19^* mutation impacted the onset, development and/or severity of alcohol-induced gut leakiness. Similar to our previous study [14], we found that alcohol caused significant intestinal hyperpermeability (Figure 3A; p<0.05, two-way ANOVA). We also found that circadian disruption led to earlier onset and greater magnitude of alcohol-induced gut leakiness: alcohol-fed *Clock^Δ19/Δ19^* mutant mice exhibited significantly elevated urinary sucralose levels compared to alcohol-fed wild-type mice that were evident after the first week on the full concentration of alcohol in the diet and remained significantly elevated after four weeks (Figure 3A; p<0.05, two-way ANOVA with *post-hoc* Tukey-Kramer Multiple-Comparison test).

The alcohol-induced increase in intestinal permeability was greater in *Clock^Δ19/Δ19^* mutant mice than in wild-type littermates, and occurred despite a significant effect of genotype on food intake (F_(1,47)_ = 4.707, p<0.05), whereby *Clock^Δ19/Δ19^* mutant mice consumed less than wild-type littermates, after correction for differences in body weight (Figure 3B). Despite the absence of increased intake, alcohol-fed *Clock^Δ19/Δ19^* mutant mice exhibited a significant elevation in serum alcohol levels (BAL) compared to alcohol-fed wild-types (Figure 3C; p<0.01, two-way ANOVA with *post-hoc* Tukey-Kramer Multiple-Comparison test). This finding may be explained, at least in part, by increased alcohol intake by *Clock^Δ19/Δ19^* mutants during the light phase (specifically, in the period immediately preceding the blood collection for measurement of BAL at ZT6) due to altered diurnal feeding rhythms in *Clock^Δ19/Δ19^* mutants [28]. Indeed, in a separate group of mice using the same chronic alcohol protocol, *Clock^Δ19/Δ19^* mutant mice consumed a significantly greater amount of the alcohol-containing diet than alcohol-fed wild-types in the two hour window between the time the diet was provided to the mice every day (ZT4) and the time tissues were harvested for analyses (ZT6): *Clock^Δ19/Δ19^* mutant alcohol  = 2.77±0.15 mL diet consumed (n = 5) *vs.* wild-type alcohol  = 2.05±0.20 mL diet consumed (n = 5); p<0.05, Student's t-test.

**Figure 4:**
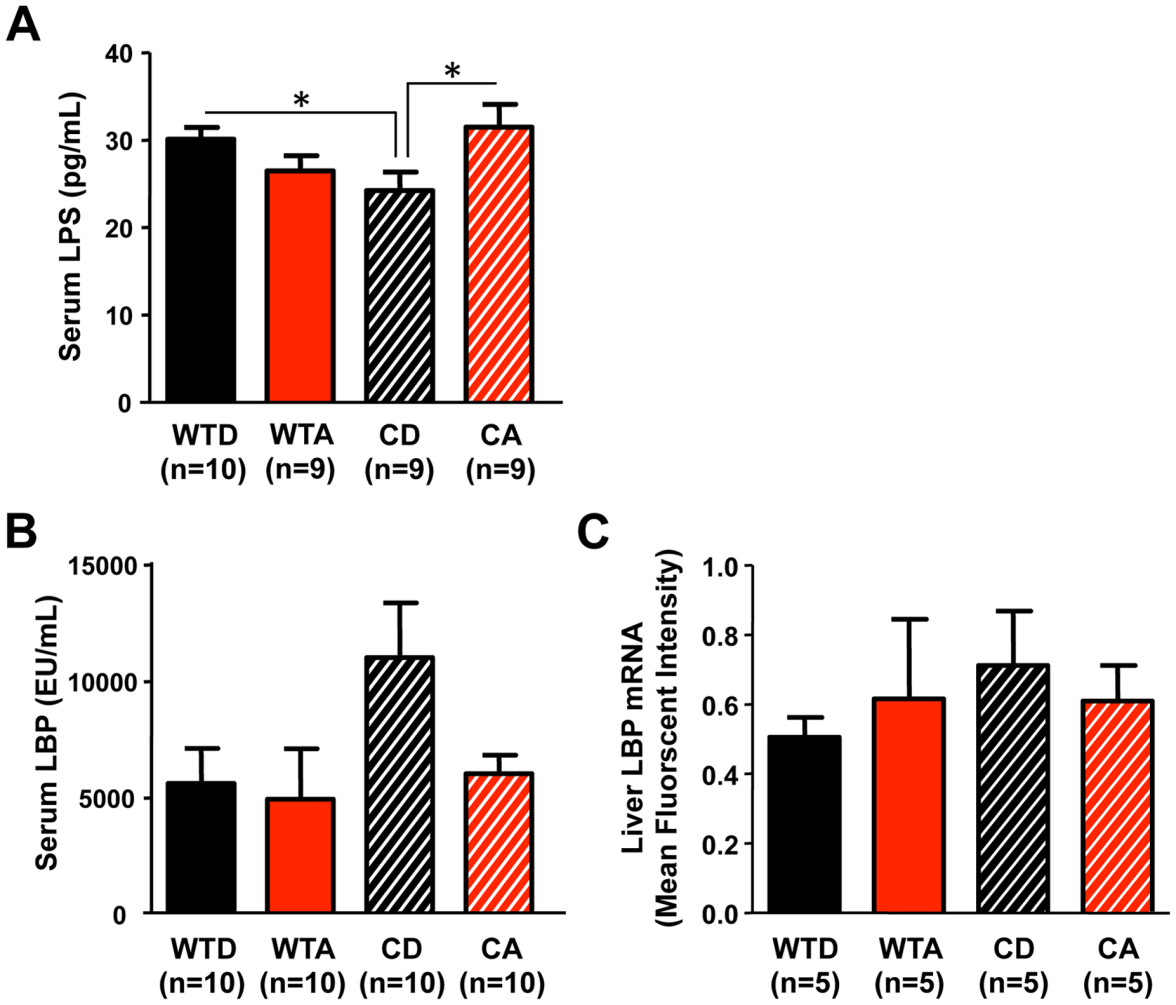
Serum lipopolysaccharide (LPS) levels are altered and affected by alcohol in *Clock^Δ19/Δ19^* mutant mice. (**A**) Compared to wild-type littermates (WTD), *Clock^Δ19/Δ19^* mutant mice on the control diet (CD) exhibited significantly reduced serum LPS levels. *Clock^Δ19/Δ19^* mutant mice on alcohol (CA) had significantly elevated serum LPS compared to mutants on the control diet (CD), with an overall significant genotype × diet interaction observed (p<0.01). *p<0.05, two-way ANOVA followed by *post-hoc* Tukey-Kramer Multiple-Comparison. There were no significant differences in serum LPS binding protein (LBP) levels (**B**) or liver LBP mRNA expression (**C**).

Intestinal hyperpermeability permits the translocation of proinflammatory bacterial products from the lumen of the intestine into systemic circulation. Most prominently, lipopolysaccharide (LPS), a component of the cell wall of Gram negative bacteria, can leak across the intestinal barrier and elicit a strong proinflammatory response. Thus, to assess the biological consequence of increased intestinal permeability in alcohol-fed *Clock^Δ19/Δ19^* mutant mice, we measured serum LPS and LPS binding protein (LBP), an acute phase protein produced by the liver that binds circulating LPS. Compared to control-fed (i.e., alcohol-free) wild-type littermates, control-fed *Clock^Δ19/Δ19^* mutant mice had significantly reduced circulating LPS levels, whereas alcohol-fed *Clock^Δ19/Δ19^* mutant mice had significantly elevated LPS (Figure 4A; p<0.05, two-way ANOVA with *post-hoc* Tukey-Kramer Multiple-Comparison test), as predicted by the increased intestinal permeability in the alcohol-fed mutant mice. Although there was a significant genotype x diet interaction (F_(1,37)_ = 7.670, p<0.001), no significant differences in serum LPS levels were observed between alcohol-fed and control-fed wild-type mice (Figure 4A). No significant between group differences in serum LBP protein or liver LBP mRNA levels were observed (Figures 4B–C).

**Figure 5:**
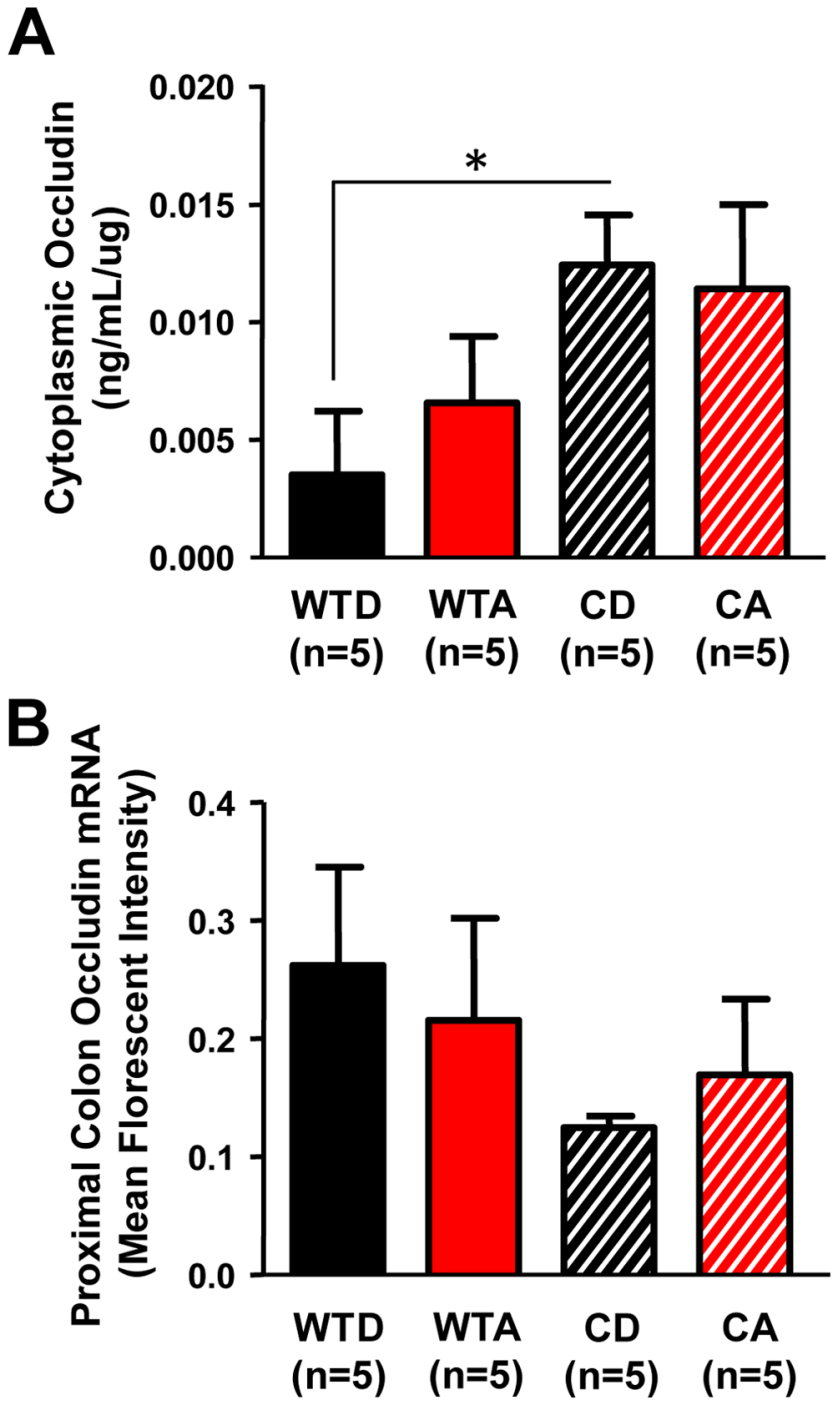
Cytoplasmic tight junction protein occludin levels in the proximal colon are significantly elevated in *Clock^Δ19/Δ19^* mutant mice. (**A**) *Clock^Δ19/Δ19^* mutant mice on the control diet (CD) had significantly elevated levels of cytoplasmic occludin in the proximal colon compared to wild-type littermates on the control diet (WTD), with an overall significant effect of genotype (p<0.05). *p<0.05, two-way ANOVA followed by *post-hoc* Fisher's LSD Multiple-Comparison test. (**B**) *Occludin* mRNA was not significantly altered.

The maintenance of intestinal barrier integrity requires a number of tight junction and apical junction proteins. One such protein, occludin, correlates with changes in intestinal permeability, with decreased expression or decreased membrane-bound (i.e., increased cytoplasmic) protein associated with increased permeability (for review, see [45] and references therein). We examined occludin in the proximal colon as a possible mechanism contributing to the promotion of intestinal permeability by circadian disruption. There was a significant effect of genotype (F_(1,20)_ = 5.83, p<0.05), with *Clock^Δ19/Δ19^* mutants having increased cytoplasmic occludin, indicative of internalized, non-membrane bound protein. *Post-hoc* analysis revealed a significant difference between control diet-fed *Clock^Δ19/Δ19^* mutants and control-fed wild-types (Figure 5A; p<0.05, two-way ANOVA with *post-hoc* Fisher's LSD Multiple-Comparison test). No significant differences in occludin mRNA expression in the proximal colon were observed (Figure 5B), suggesting that changes in protein localization may be due to a post-translational mechanism(s).

**Figure 6:**
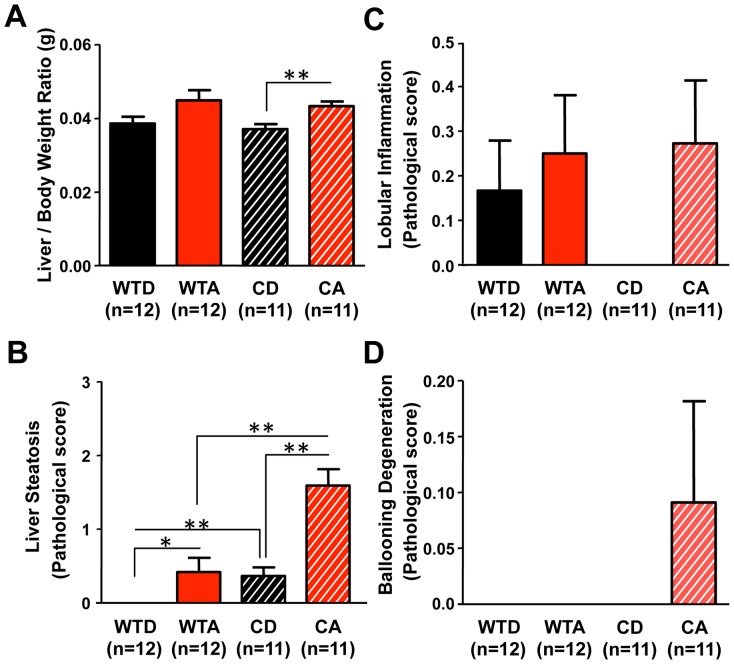
Genetic disruption of circadian organization promotes alcohol-induced hepatic steatosis. (**A**) Alcohol-fed *Clock^Δ19/Δ19^* mutant mice (CA) had a significantly greater liver/body weight ratio than control-fed mutants (CD), with an overall significant effect of diet (p<0.01). **p<0.01, two-way ANOVA followed by *post-hoc* Tukey-Kramer Multiple-Comparison test. (**B**) Alcohol-fed *Clock^Δ19/Δ19^* mutant mice (CA) exhibited significant steatosis compared to all other experimental groups, with a significant overall effect of genotype (p<0.001), diet (p<0.001), as well as a significant interaction (p<0.05). *p<0.05, **p<0.01; two-way ANOVA followed by *post-hoc* Tukey-Kramer Multiple-Comparison test. No between group differences were observed for histological assessment of lobular inflammation (**C**) or ballooning degeneration (**D**). Histological assessments were performed by a blinded gastrointestinal pathologist. Steatosis score was based on % hepatocyte involvement: 0 = <5%, 1 = 5–33%, 2 = 34–66%, 3 = >67%. Lobular inflammation score was based on the number of foci/200× field: 0 = none, 1 = 1, 2 = 2–4, 3 = >4. Ballooning degeneration score was based on the presence and frequency of ballooned cells: 0 = none, 1 = few, 2 = prominent/many.

Alcohol-fed *Clock^Δ19/Δ19^* mutant mice had a significantly higher liver/body weight ratio than control-fed mutants (Figure 6A; p<0.05, two-way ANOVA with *post-hoc* Tukey-Kramer Multiple-Comparison test), with a significant overall effect of diet observed (F_(1,46)_ = 10.222, p<0.01). In addition, alcohol-fed *Clock^Δ19/Δ19^* mutant mice had significantly increased liver steatosis compared to alcohol-fed wild-type mice and control diet-fed *Clock^Δ19/Δ19^* mutants (Figure 6B; p<0.05, two-way ANOVA with *post-hoc* Tukey-Kramer Multiple-Comparison test), with significant overall effects of genotype (F_(1,46)_ = 24.011, p<0.001) and diet (F_(1,46)_ = 27.437, p<0.05), as well as a significant genotype x diet interaction (F_(1,46)_ = 6.671, p<0.05). There were no significant between group differences in inflammation (Figure 6C), and acidophil bodies were absent in all groups (data not shown), but hepatocyte ballooning (a marker of hepatocyte injury) was only observed in alcohol-fed *Clock^Δ19/Δ19^* mutant mice (Figures 6D).

### Circadian Disruption – Environmental Model

**Figure 7:**
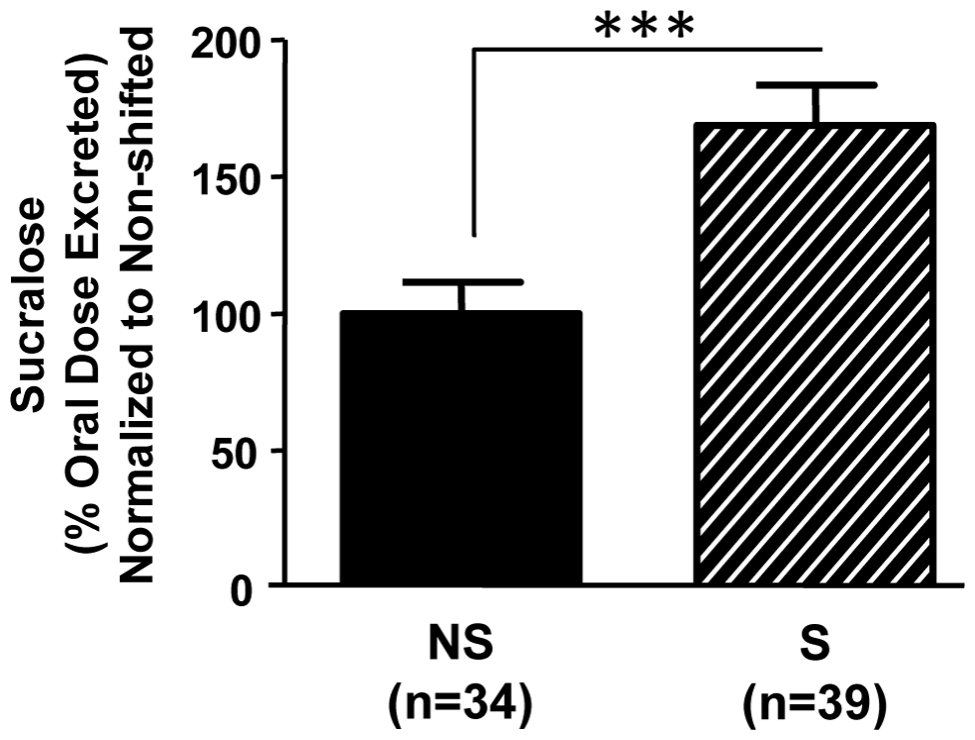
Environmental disruption of circadian organization increases intestinal permeability. Young adult (7–9 week) male C57BL/6J mice underwent three months of weekly 12 hour phase shifts of the LD cycle (S) or maintained on a constant 12∶12 LD cycle (NS). Shifted animals had significantly greater intestinal permeability. ***, p<0.001, Student's *t*-test.

We sought to determine whether environmental disruption of circadian organization in mice with genetically intact circadian clocks would also disrupt intestinal barrier function and/or promote environmentally-induced gut leakiness. To establish chronic environmental circadian disruption, mice were subjected to weekly 12 hour phase shifts of the LD cycle for three months [9]. Compared to non-shifted animals, shifted mice exhibited significantly increased intestinal permeability (Figure 7; p<0.001, Student's t-test). Five hour urinary sucralose levels were significantly higher in shifted mice (0.908±0.1% oral dose excreted) than non-shifted mice (0.538±0.1% oral dose excreted). However, there were no significant differences in urinary sucralose, mannitol or lactulose between shifted and non-shifted mice (data not shown), indicating that, similar to genetic disorganization, environmental disruption of circadian organization primarily impacts colonic permeability.

**Figure 8:**
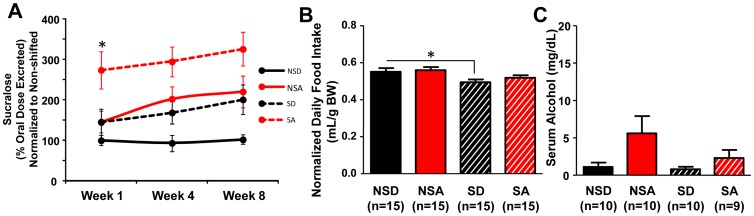
Environmental disruption of circadian organization augments alcohol-induced intestinal hyperpermeability. (**A**) Alcohol-fed shifted mice (SA, n = 13–15/week) exhibited significantly increased intestinal permeability compared to alcohol-fed non-shifted mice (NSA, n = 13–15/week), control-fed shifted mice (SD, n = 11–12/week) and control-fed non-shifted mice (NSD, n = 8–13/week). Overall significant effects of diet (p<0.01) and schedule (shifted *vs.* non-shifted, p<0.05) were observed at each time point. *, p<0.05, Tukey-Kramer Multiple-Comparison test. (**B**) Average daily diet intake (normalized to body weight) for the duration of the experiment in all groups. Shifted control-fed mice (SD) consumed less than non-shifted mice (NSD). Shifted mice (SA) did not consume more alcohol than non-shifted mice (NSA). *p<0.05, two-way ANOVA followed by *post-hoc* Tukey-Kramer Multiple-Comparison test. (**C**) There was a significant effect of diet on serum alcohol levels (F_(1,39)_ = 4.986, p<0.05). Two-way ANOVA.

As with the *Clock^Δ19/Δ19^* genetic model, phase-shifting the LD cycle on a chronic basis (Figure 1B) caused intestinal hyperpermeability (Figure 7) and increased alcohol-induced gut leakiness, resulting in earlier onset and more severe gut leakiness. Compared to non-shifted alcohol-fed mice, shifted alcohol-fed mice had significantly increased urinary sucralose levels that were evident after one week on the full alcohol diet (Figure 8A; p<0.05, two-way ANOVA with *post-hoc* Tukey-Kramer Multiple Comparison test). Interestingly, the shifted control-fed mice had intestinal permeability levels similar to the non-shifted alcohol-fed mice (Figure 8A), suggesting that chronic environmental circadian disruption alone is sufficient to increase intestinal permeability to levels comparable to those that occur as a consequence of chronic alcohol consumption.

The intestinal hyperpermeability observed in mice subjected to chronic circadian disruption was not due to increased alcohol intake (Figure 8B). There was a significant effect of schedule (i.e., shifted *vs*. non-shifted; F_(1,60)_ = 8.418, p<0.01) on diet intake, with *post-hoc* significance indicating a decrease in consumption in control-fed shifted compared to control-fed non-shifted mice (Figure 8B; p<0.05, two-way ANOVA with *post-hoc* Tukey-Kramer Multiple-Comparison test). Analysis of serum alcohol concentrations revealed a significant effect of diet (F_(1,39)_ = 4.986, p<0.05), with higher levels in alcohol-fed mice. No significant difference in serum alcohol levels was observed between alcohol-fed shifted and alcohol-fed non-shifted mice (Figure 8C).

**Figure 9:**
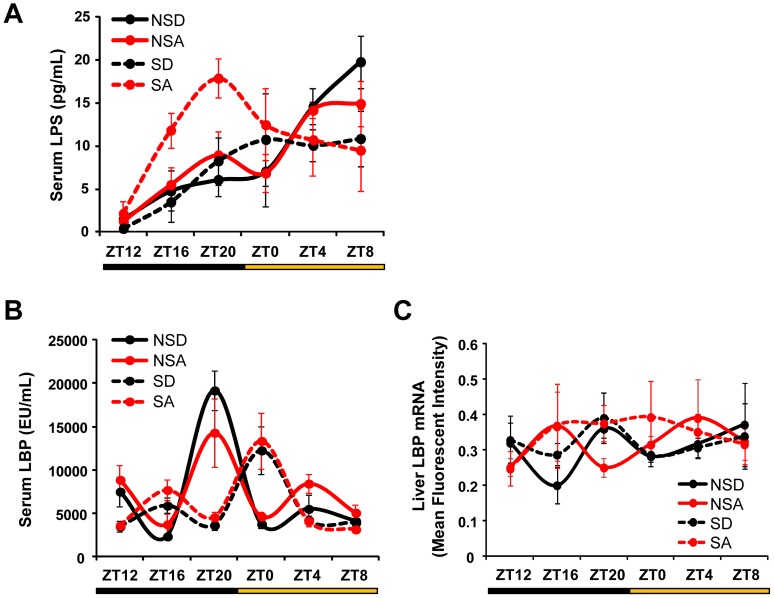
Environmental disruption of circadian organization impacts serum LPS and LBP levels. (**A**) Serum LPS levels exhibited a significant time-of-day effect (p<0.001), with a significant schedule (i.e., shifted *vs*. non-shifted) × time-of-day interaction (p<0.01). Three-way ANOVA. (**B**) There was an apparent delayed peak in serum LBP of shifted mice, independent of diet, that failed to reach statistical significance. (**C**) LBP mRNA expression in liver was not significantly affected by schedule or alcohol.

Serum LPS, LBP and liver LBP mRNA were measured to assess the biological impact of increased intestinal permeability. There was a significant time-of-day effect on serum LPS (Figure 9A; F_(1,116)_ = 10.61, p<0.001) and LBP (Figure 9B; F_(1,95)_ = 5.47, p<0.001), and there was also a significant schedule (i.e., shifted *vs*. non-shifted) x time-of-day interaction for both LPS (F_(1,116)_ = 3.30, p<0.01) and LBP (F_(1,95)_ = 12.71, p<0.001). Serum LBP levels were delayed by four hours in the shifted groups, independent of diet (i.e., alcohol-fed or control-fed), suggesting that chronic shifting of the LD cycle impacts circulating LBP levels for at least one week after the last phase shift occurred (mice were euthanized with serum harvested for analysis one week after the last phase shift). Liver LBP mRNA did not exhibit any time-of-day effects or between group differences (Figure 9C), suggesting that post-translational mechanisms or LBP produced outside the liver [46] may be responsible for diurnal rhythm of serum LBP.

**Figure 10:**
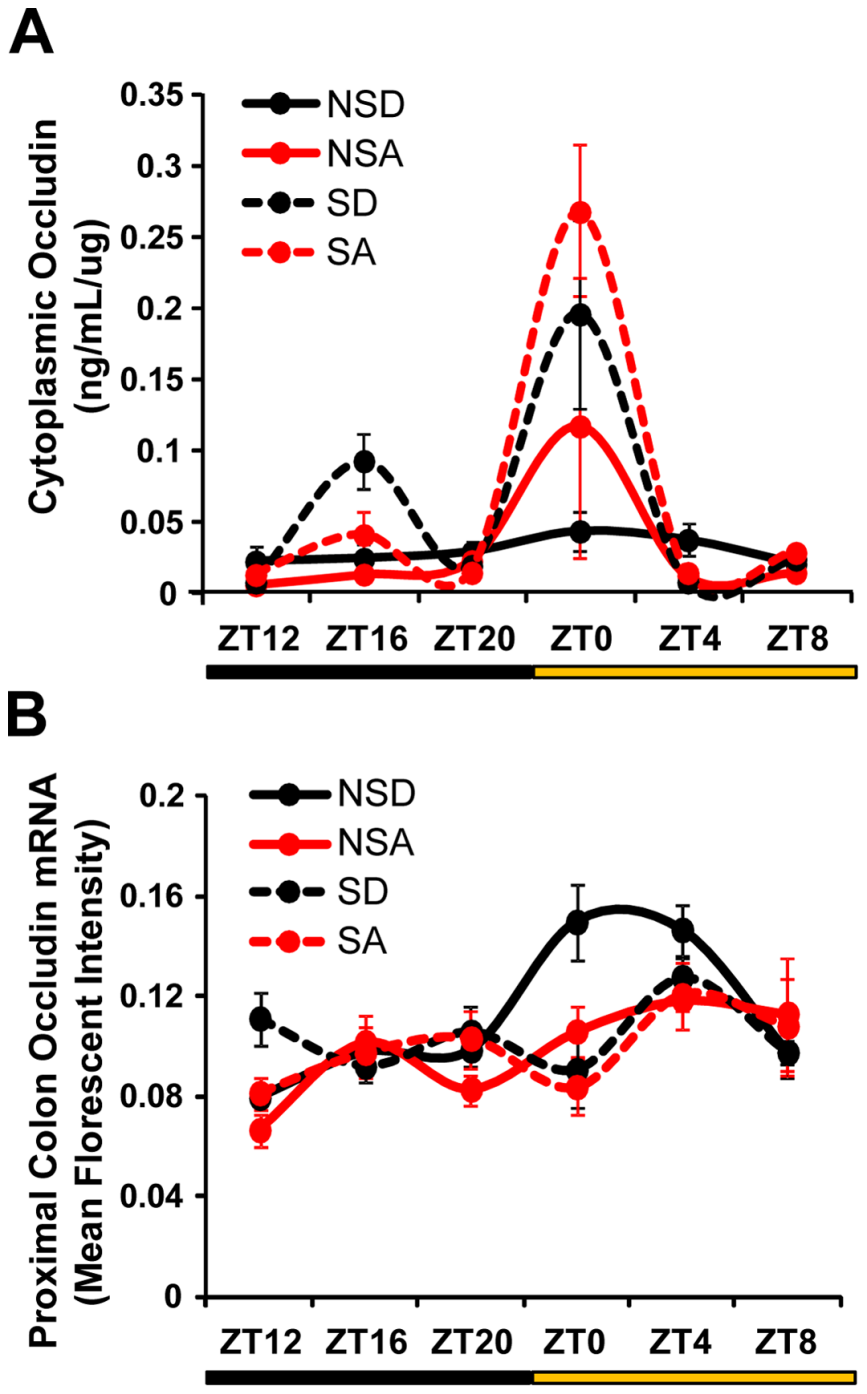
Altered regulation of the tight junction protein occludin in the proximal colon by alcohol and chronic circadian disruption. (**A**) Cytoplasmic occludin protein levels in the proximal colon were elevated at ZT0 in shifted animals (SA and SD) on both the control and alcohol diet, with an overall significant effect of schedule (i.e., shifted *vs*. non-shifted; p<0.01) and time-of-day (i.e., ZT; p<0.001). There was also a significant schedule × time-of-day interaction (p<0.001). Three-way ANOVA. (**B**) There were significant time-of-day (p<0.001) and diet (p<0.05) effects on proximal colon *occludin* mRNA expression, with a significant schedule × time-of-day interaction (p<0.01). Three-way ANOVA.

Given the critical role of the tight junction protein occludin in regulating intestinal barrier integrity, we measured occludin protein and mRNA levels in the proximal colon. Cytoplasmic levels of the occludin protein were increased by both alcohol and phase-shifting of the LD cycle, with peak levels occurring at ZT0 (Figure 10A). There was a significant overall effect of time-of-day (F_(1,119)_ = 17.62, p<0.001) and schedule (i.e., shifted vs. non-shifted; F_(1,119)_ = 7.76, p<0.001), as well as a significant schedule × time-of-day interaction (F_(1,119)_ = 5.81, p<0.001). Occludin mRNA levels were also significantly affected by time-of-day (F_(1,117)_ = 7.44, p<0.001) and diet (F_(1,117)_ = 4.53, p<0.05), and there was a significant schedule × time-of-day interaction (F_(1,117)_ = 4.47, p<0.01); however there were no significant between group differences in mRNA expression of occludin in the proximal colon (Figure 10B), suggesting that the increased cytoplasmic occludin protein levels seen in the alcohol-fed and shifted mice may be due to post-translational mechanisms, possibly involving subcellular localization.

**Figure 11:**
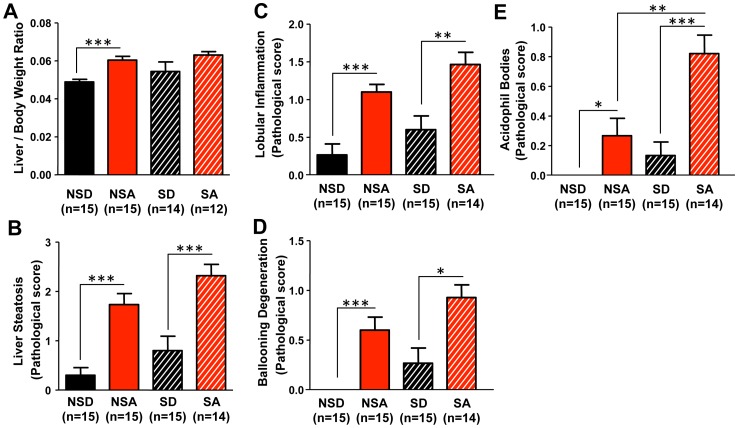
Environmental disruption of circadian organization promotes alcohol-induced liver pathology. (**A**) There was an overall effect of diet for the liver/body weight ratio (p<0.001). The ratio was significantly elevated in non-shifted alcohol-fed mice (NSA) compared to non-shifted control-fed mice (NSD). There was a trend for increased liver/body weight ratio in alcohol-fed shifted mice (SA) compared to control-fed shifted mice (SD), although the difference failed to reach statistical significance. ***p<0.001, two-way ANOVA followed by *post-hoc* Tukey-Kramer Multiple-Comparison test. Histological assessment of liver steatosis (p<0.001) (**B**), lobular inflammation (p<0.001) (**C**), ballooning degeneration (p<0.001) (**D**) and the presence of acidophil bodies (p<0.001) (**E**) all revealed significant effects of diet, with ballooning degeneration (p<0.05) and acidophil bodies (p<0.05) also demonstrating significant effects of schedule (i.e, shifted *vs*. non-shifted). *p<0.05, **p<0.01, ***p<0.001; two-way ANOVA followed by *post-hoc* Tukey-Kramer Multiple-Comparison test. Histological assessment of liver samples was performed by a blinded gastrointestinal pathologist. Steatosis score was based on % hepatocyte involvement: 0 = <5%, 1 = 5–33%, 2 = 34–66%, 3 = >67%. Lobular inflammation score was based on the number of foci/200× field: 0 = none, 1 = 1, 2 = 2–4, 3 = >4. Ballooning degeneration score was based on the presence and frequency of ballooned cells: 0 = none, 1 = few, 2 = prominent/many. Acidophil body score was based on the presence of acidophil bodies: 0 = absent, 1 = focal apoptosis (few), 2 = many, 3 = confluent necrosis.

Analysis of gross liver fat content, as assessed by the liver weight/body weight ratio, was undertaken as an indication of pathological hepatic transformation. A significant overall effect of diet was observed (F_(1,56)_ = 11.820, p<0.01), with alcohol-fed mice having a higher liver/body weight ratio. *Post-hoc* analysis revealed a significant increase non-shifted alcohol-fed compared to non-shifted control-fed mice (Figure 11A; p<0.05, two-way ANOVA with *post-hoc* Tukey-Kramer Multiple-Comparison test). The histological measures of liver pathology revealed an alcohol-induced increase in steatosis (Figure 11B, F_(1,60)_ = 34.612, p<0.001), lobular inflammation (Figure 11C, F_(1,60)_ = 26.396, p<0.001), ballooning degeneration (Figure 11D, F_(1,59)_ = 28.152, p<0.001) and the presence of acidophil bodies (Figure 11E, F_(1,59)_ = 24.727, p<0.001), with significant overall effects of schedule for ballooning degeneration (Figure 11D; F_(1,59)_ = 6.264, p<0.05) and acidophil body presence (Figure 11E; F_(1,50)_ = 17,843, p<0.001). For all measurements, the shifted alcohol-fed mice exhibited worsened pathological damage and inflammation, although this difference reached *post-hoc* statistical significance only for acidophil bodies (Figure 11E; p<0.01, two-way ANOVA with *post-hoc* Tukey-Kramer Multiple-Comparison test). Taken together, these results suggest that environmental disruption of circadian organization augments alcohol-induced hepatic pathology *via* a mechanism involving increased intestinal permeability.

## Discussion

Here we report, for the first time, that circadian disorganization causes increased permeability of the intestinal epithelial barrier in mice. Both genetic and environmental strategies of circadian disruption result in gut leakiness and promote alcohol-induced intestinal hyperpermeability, endotoxemia and liver steatosis. Phase-shifting of the LD cycle also increases hepatic cell injury in response to chronic alcohol consumption. These results indicate that circadian organization has a critical function in the maintenance of intestinal barrier integrity and suggest that circadian disruption may be a previously unrecognized risk factor for alcohol-induced liver injury.

The circadian clock imposes temporal organization to ongoing physiological and biochemical processes at multiple levels, thus optimizing function and, presumably, enhancing fitness. Therefore, circadian disruption is expected to result in adverse physiological consequences detrimental to the health and well-being of the organism. Indeed, a rapidly accumulating body of evidence supports this hypothesis. In humans, shift workers are known to be at increased risk for a number of chronic diseases and cardiometabolic abnormalities [47], [48], [49], [50]. In rodents, animals harboring mutations of core circadian clock genes exhibit multiple physiological and behavioral abnormalities [28], [29], [51], [52], [53], [54]. Chronic circadian disruption, achieved with repeated exposure to phase shifts of the LD cycle, accelerates and/or exacerbates numerous pathologies in rodents [9], [55], [56], and increases mortality in hamsters with a genetic predisposition to cardiomyopathy [56] and in aged mice [57]. These studies, as well as others, support a model in which the adverse effects of circadian disruption become evident in the presence of a physiological “challenge,” such as a genetic predisposition to disease [56], aging [57], a high-fat diet [28], pregnancy [25] or chemically-induced colitis [9]. Thus, it may be appropriate to consider circadian disruption as a “second hit” that can promote disease in susceptible individuals.

Interestingly, most, if not all, pathologies associated with disrupted circadian organization share non-pathogen mediated inflammation as a common characteristic. For example, there is substantial evidence from studies in humans and in rodent models, linking circadian disruption and metabolic syndrome [58], [59], a constellation of abnormalities characterized, at least in part, by inflammation [60], [61]. Recently, it was shown that exposure to chronic phase shifts of the LD cycle in mice caused significant alteration and dysregulation of immune and inflammatory responses, particularly involving peritoneal macrophages that respond to LPS [55], however the mechanism(s) of circadian disruption-induced inflammation is not well-established.

A potential source of and trigger for such inflammation is the intestine and the microbiota. The intestinal epithelial barrier serves as the interface between the luminal proinflammatory microbiota and the immune system [11]. The microbiota influences immune regulation throughout the gut [11], and alterations of intestinal microbiota have been associated with intestinal and systemic inflammation [62]. The intestinal epithelial barrier regulates the exposure of proinflammatory luminal contents, such as endotoxin, to immune cells and systemic circulation, thus controlling the local and systemic inflammatory responses to gut-derived endotoxin. While a small degree of intestinal permeability is physiological, presumably to enable immune surveillance and regulation [11], increases in permeability have been associated with numerous proinflammatory pathological states [12], [13], [14], [15], [16]. Given the prominent role of circadian rhythms in the regulation of gastrointestinal physiology in mammals [63], the aim of the current study was to test the hypothesis that altered circadian organization impairs intestinal barrier integrity.

The evidence supporting this hypothesis comes from studies demonstrating that chronic circadian disruption in mice (achieved using repeated exposure to phase shifts of the LD cycle) increases susceptibility to DSS-induced colitis [9]. Intestinal epithelial cells are the target of DSS [64], suggesting that chronic circadian disruption may impair or sensitize to injury the cells that form and regulate the intestinal barrier. Also, studies of cultured human intestinal epithelial cells (i.e., Caco-2 cells) indicate that the expression of circadian clock genes contributes to alcohol-induced intestinal hyperpermeability *in vitro* [22]. Although supportive, these lines of evidence are indirect, thus an *in vivo* model is required, and to achieve this, a reliable and repeatable measure of intestinal permeability is necessary. We measured the urinary excretion of orally administered, non-metabolized, non-absorbed sugars. Four sugars were utilized (sucrose, sucralose, mannitol and lactulose) in order to assess the location in the gastrointestinal tract that leakiness occurs: sucrose for gastroduodenal (0–3 hours), mannitol and lactulose for small bowel (3–5 hours) and sucralose for colon (5 hours) [65]. We observed increased permeability to sucralose (Figures 2 and 7), but not sucrose, mannitol or lactulose (data not shown), enabling us to conclude that circadian disruption causes increased permeability, which occurs predominantly in the colon.

Notably, both genetic and environmental approaches of disruption resulted in increased intestinal permeability, strongly supporting a critical role for circadian organization in maintaining intestinal barrier integrity. Circadian disorganization caused gut leakiness whether it was present throughout the life of the organism (i.e., genetic model) or if it was induced in adulthood (i.e., environmental model) and promoted alcohol-induced gut leakiness (Figures 3 and 8). Interestingly, the impact of phase shifting the LD cycle alone on intestinal permeability was similar in magnitude to that caused by alcohol consumption (Figure 8), indicating that circadian disruption can induce as much as gut leakiness as alcohol, a well-known risk factor for increased intestinal permeability [14], [15].

After establishing the role of circadian disruption in promoting gut leakiness, we sought to assess the biological impact. In order to test this, a model system wherein gut leakiness is central to tissue injury is required. Thus, we chose chronic alcohol consumption, as we have previously shown that increased intestinal permeability precedes the development of alcoholic steatohepatitis in a rat model [14]. Liver injury is known to be one of the most common and serious side effects of chronic alcohol consumption [66], [67], [68], and inflammation is a key driver of alcohol-induced hepatic pathology [69], [70], [71], [72]. Consistent with the observed increase in intestinal permeability, we found that *Clock^Δ19/Δ19^* mutant mice fed alcohol exhibit significant endotoxemia compared to mutants on the control diet (Figure 4A), and environmentally disrupted alcohol-fed mice have significantly altered LPS rhythms in the serum (Figure 9A).

Interestingly, *Clock^Δ19/Δ19^* mutant mice on the control diet had a significant reduction in serum LPS levels compared to wild-type littermates on the control diet (Figure 4A). Perhaps this reflects a compensatory mechanism in *Clock^Δ19/Δ19^* mutant mice that have increased intestinal permeability and endotoxin exposure throughout their lives. There was a reduction in serum LBP levels in alcohol-fed *Clock^Δ19/Δ19^* mutants compared to those on the control diet (Figure 4B). The assay measures free LBP in the serum, thus a reduction in LBP levels in the alcohol-fed *Clock^Δ19/Δ19^* mutants likely reflects an increase in LBP bound to LPS. We observed a significant time-of-day effect for serum LPS and LBP (Figure 9A–9B), similar to the LPS rhythm that has been observed as a consequence of consuming a high-fat diet [73]. This rhythm has clinical and translational implications, as it highlights the importance of the timing of blood sample collection as a critical variable to consider when measuring serum LPS and drawing conclusions about endotoxemia. Alcohol and phase shifts of the LD cycle advanced the phase of the serum LPS rhythm (Figure 9A). We observed a phase delay in the rhythm of serum LBP in shifted mice independent of diet (Figure 9B), suggesting that the impact of the phase shifts was greater than that of alcohol. We did not observe a rhythm of LBP expression in the liver (Figure 9C), suggesting that post-translational mechanisms may contribute to the diurnal rhythm in protein levels observed in the serum, or that LBP production in other organs (e.g., the lung [46]) may be contributing to serum LBP regulation.

In addition to the increase in endotoxin, alcohol-fed *Clock^Δ19/Δ19^* mutant mice exhibited a significantly elevated liver/body weight ratio (Figure 6A) and promoted alcohol-induced liver steatosis (Figure 6B). These results are consistent with a previous report of increased steatosis in alcohol-fed *Clock^Δ19/Δ19^* mutant mice [74] and suggest that increased permeability and endotoxemia are associated with increased steatosis in alcohol-fed *Clock^Δ19/Δ19^* mutant mice. However, except for a trend for increased ballooning degeneration in alcohol-fed *Clock^Δ19/Δ19^* mutants (Figure 6D), there was no evidence of increased inflammation or hepatocyte injury (Figure 6C, 6D). This result may be the consequence of altered immune regulation caused by the *Clock^Δ19^* mutation and/or a compensatory mechanism, such as endotoxin tolerance [75], due to increased intestinal permeability in *Clock^Δ19/Δ19^* mutant mice since early in life. Interestingly, *Clock^Δ19/Δ19^* mutant mice exhibited a significant elevation of serum alcohol levels compared to alcohol-fed wild-type mice (Figure 3C), despite the fact that *Clock^Δ19/Δ19^* mutant mice did not consume more alcohol (Figure 3B), which has been observed in mice harboring a mutation in the circadian clock gene *Per2* [76]. Although we observed increased food consumption in *Clock^Δ19/Δ19^* mutant mice in the two hour window preceding blood collection, which may contribute to the observed elevation in serum alcohol levels, we cannot exclude the possibility that alcohol metabolism was altered or impaired in *Clock^Δ19/Δ19^* mutant mice.

The alcohol-fed shifted mice exhibited significant elevations in steatosis (Figure 11B), lobular inflammation (Figure 11C) and hepatocyte injury (Figure 11D–11E). These indicators of alcohol-induced hepatic pathology were all greater in the alcohol-fed shifted mice compared to non-shifted mice, although the difference reached statistical significance only for the presence of acidophil bodies. These histological findings demonstrate that phase shifts of the LD cycle increase hepatocyte injury induced by alcohol, as evidenced by the inflammation and hepatocyte injury (i.e., hepatocyte ballooning and the presence of acidophil bodies). Further studies are necessary to determine if the phase shifts of the LD cycle increase hepatocyte apoptosis. Taken together, these findings implicate circadian disruption as a factor capable of promoting alcohol-induced hepatic injury and pathology, consistent with our hypothesis that circadian disruption augments alcoholic-induced intestinal hyperpermeability, endotoxemia, inflammation and hepatic injury.

A potential mechanism underlying the impact of circadian disruption on intestinal permeability involves the tight junction protein occludin, the expression of which has been correlated with permeability, with lower membrane-bound protein associated with increased leakiness [45]. Cytoplasmic occludin levels were higher (i.e., membrane-bound levels were reduced) in alcohol- and control-fed *Clock^Δ19/Δ19^* mice (Figure 5A). The similarity between alcohol- and control-fed *Clock^Δ19/Δ19^* mutants suggests that occludin localization may be entirely due to the *Clock^Δ19^* mutation, or that there is a ceiling effect that cannot be augmented by alcohol. Similarly, chronic phase shifting of the LD cycle, in conjunction with alcohol, induced a cytoplasmic localization rhythm of occludin, with a peak at ZT0 (Figure 10A). This time point coincides with the start of the intestinal permeability assessment, suggesting that the difference in occludin localization may account, at least in part, for the increase in intestinal permeability in the alcohol-fed and shifted mice. These differences in occludin protein localization occur despite similar occludin mRNA levels in the colon (Figure 10B), highlighting post-translational modification, particularly those affecting subcellular localization, as a factor underlying the observed rhythm in cytoplasmic occludin levels in alcohol-fed and shifted mice. Further studies are necessary to determine the role of circadian regulation on the trafficking of tight junctional proteins.

Another potential mechanism underlying the impact of circadian disruption on intestinal hyperpermeability, endotoxemia and inflammatory hepatic pathology that we cannot exclude at present is possible changes to the microbiota induced by circadian disruption. While it has been established that chronic alcohol leads to dysbiosis [77], [78], [79], [80], [81], the effects of circadian disorganization on the composition of the microbiota are not known.

Based on the results presented here, we hypothesize that disrupted circadian organization is a previously unrecognized risk factor for alcoholic liver disease (ALD). The identification of circadian disruption as an ALD risk factor in humans would open up a new avenue for risk stratification, risk reduction, prevention and/or treatment. Indeed it is not clear why only a subset of alcoholics is susceptible to the development of severe liver (and other organ) pathology [82], and disrupted circadian organization may be a factor contributing to the differential susceptibility. The establishment of circadian disruption as a contributor to increased intestinal permeability would have clear scientific and clinical implications given the importance of intestinal barrier integrity in maintaining health not only for intestinal function, but also for other diseases (e.g., Parkinson's disease [13] and metabolic diseases [12]) that have been associated with excessive alcohol intake and/or gut leakiness.
